# Availability of consent forms in ClinicalTrials.gov for industry-sponsored trials

**DOI:** 10.1093/haschl/qxaf219

**Published:** 2025-11-13

**Authors:** Peter Doshi, Jerry Menikoff, Robert Morlock, Michael Wilkes, Deborah Zarin, John H Powers

**Affiliations:** Department of Practice, Sciences, and Health Outcomes Research, University of Maryland School of Pharmacy, Baltimore, MD 21201, United States; Centre for Biomedical Ethics, Yong Loo Lin School of Medicine, National University of Singapore, Singapore 129793, Singapore; Health Services Research, Acumen Health Research Institute, Ann Arbor, MI 48104, United States; Department of Internal Medicine, School of Medicine, University of California, Davis, CA 95616, United States; Chevy Chase, MD 20815, United States; Department of Practice, Sciences, and Health Outcomes Research, University of Maryland School of Pharmacy, Baltimore, MD 21201, United States; Department of Medicine, George Washington University School of Medicine, Washington, DC 20052, United States

## Introduction

Informed consent forms (ICFs) are critical to obtaining informed consent for research studies. ICFs should clearly explain a study's purpose, potential benefits, risks, and alternatives to individuals considering enrollment, enabling potential participants to make informed decisions about whether to participate, helping safeguard patient autonomy and right to self-determination.^1,2^ Unfortunately, many ICFs do not meet expectations.^3^

Publicly posting ICFs is important because transparency might encourage sponsors to create higher quality ICFs and enable the meta-research necessary to promote continuous quality improvement.^2,4-6^

As of July 2017, the ClinicalTrials.gov database enabled the voluntary posting of ICFs for any registered study. ICFs can be posted at any time during a study's lifecycle and, when relevant, multiple versions can be posted. In addition to voluntary posting, trials subject to the United States’ revised Common Rule (2017) (ie, those with US federal funding) that were approved by an institutional review board (IRB) after January 21, 2019, are required to post an ICF after trial recruitment has completed. Industry-sponsored trials without federal funding are not subject to the Common Rule.

We sought to ascertain the public availability of ICFs for industry-sponsored trials in ClinicalTrials.gov.

## Methods

We searched ClinicalTrials.gov for all industry-sponsored interventional trials without federal funding. Our search strategy utilized the Lead Sponsor Class and Funder Type fields in ClinicalTrials.gov, to identify industry-sponsored trials without federal funding as well as federally funded trials. Details of the search, conducted July 1, 2025, are provided in the table footnote.

We assessed ICF availability among the “most active” companies, defined as those sponsors with the highest number of registered clinical trials. As a comparison, we calculated the ICF posting rate among all federally funded trials subject to Common Rule reporting requirements.

Our search had no date restrictions, but to assess ICF posting behavior in the period since the Common Rule, our primary analysis focused on trials that were closed to recruitment (ie, study status was active not recruiting, completed, or terminated) with a study start date on or after January 21, 2019. (The study start date was used to approximate the date of a study’s institutional review board approval, the criteria specified by the Common Rule, but is not held in ClinicalTrials.gov. ClinicalTrials.gov does require the responsible party to state that the study was IRB approved before the trial can enter the “recruiting” phase.) Statistical analyses were conducted using Stata 17. Our dataset and code can be accessed online (https://doi.org/10.5281/zenodo.17713349).

## Results

Our sample contained 104 011 industry-sponsored interventional trials from 12 834 unique sponsors. A total of 422 ICFs were posted by 2% of the sponsors. The reporting frequency increased in the period since the January 21, 2019 cutoff date, from 0.3% to 0.6% (Table 1).

**Table 1.** ICF Reporting frequencies among all industry-sponsored and federally funded trials in ClinicalTrials.gov.

	Trials, *n*	ICFs posted, *n* (%)
Interventional trials		
Federally funded trials^a^	40 899	4396 (11%)
Start date before January 21, 2019	27 699^b^	1687 (6%)
Start date on or after January 21, 2019	12 908^b^	2709 (21%)
Industry-sponsored trials^c^	104 011	422 (0.4%)
Start date before January 21, 2019	67 162^d^	202 (0.3%)
Start date on or after January 21, 2019	36 039^d^	220 (0.6%)

^a^Interventional trials in receipt of any federal funding (ClinicalTrials.gov search string: AREA[StudyType](INTERVENTIONAL) AND AREA[FunderTypeSearch](NIH OR FED)).

^b^Study start date could not be determined for 292 trials; these studies are excluded.

^c^Interventional trials not in receipt of any federal funding with industry listed as lead sponsor (ClinicalTrials.gov search string: AREA[BasicSearch](AREA[LeadSponsorClass]INDUSTRY) AND AREA[StudyType](INTERVENTIONAL)).

^d^Study start date could not be determined for 810 trials; these studies are excluded.

Among the 3079 trials conducted by the 10 most active companies, only one company (AbbVie) posted any ICFs (1 ICF of 220 trials). The sponsors posting the most ICFs, with 4 ICFs each, were Regeneron, Janssen Vaccines, Erchonia Corporation, and Han Xu (a sponsor-investigator) (Table 2).

**Table 2.** ICF Reporting frequencies among interventional trials closed to recruitment that had a study start date on or after January 21, 2019.

	Trials, *n*	ICFs posted, *n* (%)
Federally funded trials	6018	2473 (41%)
Industry-sponsored trials	21 257	179 (0.8%)
Most active sponsors		
Pfizer	443	0 (0%)
AstraZeneca	420	0 (0%)
Eli Lilly and Company	396	0 (0%)
Merck Sharp & Dohme LLC	331	0 (0%)
Novartis Pharmaceuticals	312	0 (0%)
Boehringer Ingelheim	296	0 (0%)
Hoffman-La Roche	221	0 (0%)
Janssen Research & Development, LLC	221	0 (0%)
AbbVie	220	1 (0.5%)
GlaxoSmithKline	219	0 (0%)
Most ICFs posted^a^		
Regeneron Pharmaceuticals	86	4 (5%)
Janssen Vaccines & Prevention B.V.	23	4 (17%)
Erchonia Corporation	8	4 (50%)
Han Xu, M.D., Ph.D., FAPCR, Sponsor-Investigator, IRB Chair	6	4 (67%)
Oregon Research Behavioral Intervention Strategies, Inc.	9	3 (33%)
Theranica	4	3 (75%)
Flint Rehabilitation Devices, LLC	3	3 (100%)

^a^Of 5369 sponsors, 4 posted 4 ICFs, 3 posted 3 ICFs, 19 posted 2 ICFs, and 116 posted 1 ICF.

For all federally funded trials in ClinicalTrials.gov, ICFs were available for 11% (4396 of 40 899 trials). Among trials started since January 21, 2019, ICFs were available for 21% (2709 of 12 908), and among those also closed to recruitment, 41% (2473 of 6018) had posted an ICF (Tables 1 and 2).

## Discussion

As a result of the changes to ClinicalTrials.gov and the Revised Common Rule, there are over 12 000 ICFs in ClinicalTrials.gov today, providing a rich source of data for studying and improving informed consent processes. However, our study found that significant gaps remain, particularly among industry-sponsored trials, where ICFs are available for only a small fraction (0.6%) of recent trials. This contrasts with federally funded trials, where 21% have available ICFs.

Our study reveals the impact of required posting. Among trials closed to recruitment that started on or after January 2019, ICFs are available for 41% of federally funded trials compared to just 0.8% of industry-sponsored trials. Lawmakers and regulators should extend the requirements to include industry-sponsored trials for the same reasons that apply to federally funded trials: consent is a core element of the ethics of conducting such trials, and making consent forms public can play a major role in improving the quality of these forms. Given sub-optimal reporting for federally funded trials, increased enforcement should be considered. Although not required by the Common Rule, revised regulations could require posting ICFs during trial recruitment, improving the ability of potential participants to consult with their healthcare advisors and make better choices in deciding among multiple trial options.

## Supplementary Material

qxaf219_Supplementary_Data
