# Supplementary material for: Availability of consent forms in ClinicalTrials.gov for industry-sponsored trials
Source: Health Aff Sch. 2025 Nov 13;3(12):qxaf219. doi: 10.1093/haschl/qxaf219 (PMC12680436; doi:10.1093/haschl/qxaf219)
Supplement: qxaf219_Supplementary_Data [file qxaf219_supplementary_data.zip › Author disclosures.docx]

**Author disclosures**

**Funding & competing interests:** The authors thank Arnold Ventures for funding this study. Dr. Doshi declares grants from the FDA (through University of Maryland M-CERSI; 2020) and is Senior Editor, Investigations, The BMJ.
